# Towards Novel Non-Invasive Colorectal Cancer Screening Methods: A Comprehensive Review

**DOI:** 10.3390/cancers13081820

**Published:** 2021-04-10

**Authors:** Allegra Ferrari, Isabelle Neefs, Sarah Hoeck, Marc Peeters, Guido Van Hal

**Affiliations:** 1 Social Epidemiology and Health Policy, University of Antwerp, 2610 Antwerpen, Belgium; sarah.hoeck@bevolkingsonderzoek.be (S.H.); guido.vanhal@uantwerpen.be (G.V.H.); 2 Center for Oncological Research (CORE), University of Antwerp and Antwerp University Hospital, 2610 Antwerpen, Belgium; Isabelle.Neefs@uantwerpen.be (I.N.); Marc.Peeters@uza.be (M.P.); 3 Center of Medical Genetics, University of Antwerp and Antwerp University Hospital, 2610 Antwerpen, Belgium; 4 Center for Cancer Detection, 8000 Bruges, Belgium; 5 Department of Oncology, Antwerp University Hospital (UZA), 2650 Edegem, Belgium

**Keywords:** colorectal cancer screening, test, alternative, non-invasive, CRC, review

## Abstract

**Simple Summary:**

Since the 70s, a variety of colorectal cancer (CRC) screening programs have been adopted throughout the world with the aim of reducing the mortality rate of one of the leading cancer-related causes of death in the world. However, currently employed techniques present numerous shortcomings that negatively affect early-stage CRC detection, thus urging us to consider new and improved alternatives. Among the cited shortcomings are invasiveness and cultural stigma surrounding certain sample collection techniques, both of which negatively affect screening compliance. For this reason, many of the viable alternatives collected and described in this review aim to achieve good diagnostic performance while minimizing patient stress and discomfort. This text should serve as a guiding light for healthcare providers specialized in preventive medicine in the continuous pursuit of improved patient care.

**Abstract:**

Colorectal cancer (CRC) is one of the leading cancer-related causes of death in the world. Since the 70s, many countries have adopted different CRC screening programs, which has resulted in a decrease in mortality. However, current screening test options still present downsides. The commercialized stool-based tests present high false-positive rates and low sensitivity, which negatively affects the detection of early stage carcinogenesis. The gold standard colonoscopy has low uptake due to its invasiveness and the perception of discomfort and embarrassment that the procedure may bring. In this review, we collected and described the latest data about alternative CRC screening techniques that can overcome these disadvantages. Web of Science and PubMed were employed as search engines for studies reporting on CRC screening tests and future perspectives. The searches generated 555 articles, of which 93 titles were selected. Finally, a total of 50 studies, describing 14 different CRC alternative tests, were included. Among the investigated techniques, the main feature that could have an impact on CRC screening perception and uptake was the ease of sample collection. Urine, exhaled breath, and blood-based tests promise to achieve good diagnostic performance (sensitivity of 63–100%, 90–95%, and 47–97%, respectively) while minimizing stress and discomfort for the patient.

## 1. Introduction

From its introduction in the 70s, colorectal cancer (CRC) screening has been developing and evolving at a dramatically fast pace, with many new studies revealing potential markers for early diagnosis of CRC. Current CRC screening options, suggested by international guidelines, can be classified as either stool-based or imaging tests. The stool-based tests’ principle is that of detecting bleeding or shedding of neoplastic cells in patients’ stool. On the other hand, the aim of imaging tests is to directly visualize colonic polyps and cancers [1].

For a population between 45–80 years of age, the European guidelines for quality assurance in colorectal cancer screening and diagnosis recommend Immunochemical FOBT (iFOBT or FIT), Guaiac Fecal Occult Blood Test (gFOBT), flexible sigmoidoscopy (FSIG), and colonoscopy as current gold standard tests for screening [2]. Within the considered population (45–80), at least the age group of 60–64 should be included due to highest incidence and mortality. While these guidelines recognize newer screening technologies such as computed tomography (CT) colonography, stool DNA testing, and capsule endoscopy as emerging possibilities, they do not recommend using them for screening the average-risk population. The American Cancer Society guidelines recommend the same tests, starting at the age of 45 until the age of 75. Nevertheless, they also indicate the Multi-target Stool DNA test (MT-sDNA) and CT colonography (virtual colonoscopy) as possible options for the average-risk population [3].

However, the above-mentioned tests may present certain down sides (high false-positive rates and low sensitivity for stool-based tests; invasiveness; and the need for bowel preparation, which negatively affects the compliance for colonoscopies). In fact, the aim of this review is to describe alternative techniques in the field of CRC screening that may facilitate sample collection (e.g., blood, urine and breath-based tests), thereby positively affecting compliance or heightening sensitivity and lower false-positive rates. Because of these reasons, we believe it is important to give the reader an overview of the most promising studies on this topic.

In general, the principles of evidence-based medicine would require studies such as randomized controlled trials (RCTs) presenting mortality outcomes as the gold standard in order to demonstrate the efficacy of screening tests and preventive interventions. However, because RCTs are often not feasible, also observational studies, such as case-control designs, have been used to assess the effectiveness of colorectal cancer screenings [4] and are consequentially reported in this literature review.

## 2. Materials and Methods

**Figure 1:**
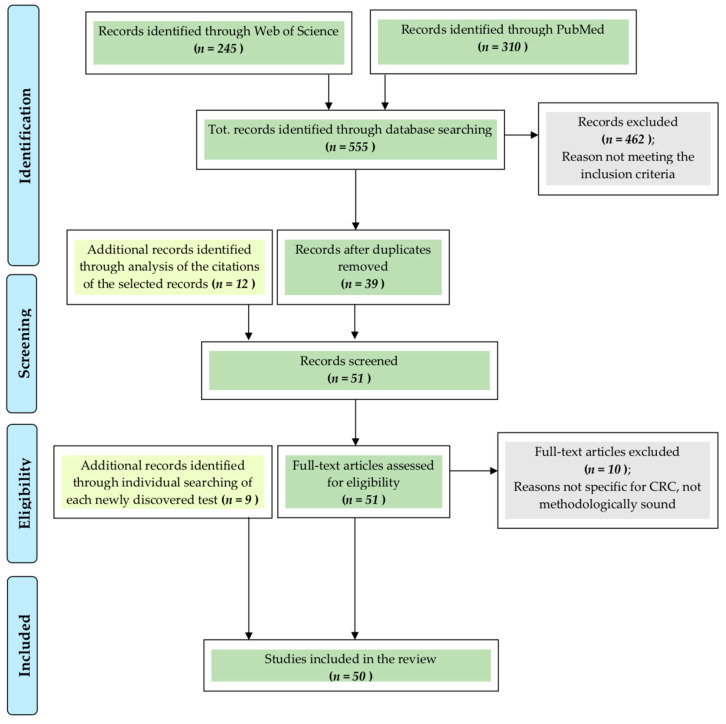
Methods. CRC = colorectal cancer.

Adapted from: Moher, D.; Liberati, A.; Tetzlaff, J.; Altman, D.G.; The PRISMA Group Preferred Reporting Items for Systematic Reviews and Meta-Analyses: The PRISMA Statement. PLoS Med **2009**, *6*, e1000097. doi:10.1371/journal.pmed1000097 [6].

As illustrated in Figure 1, in Web of Science and PubMed studies reporting on colorectal cancer, screening tests and future perspectives were searched with following inclusion criteria: I. articles written in English, II. articles from the last 5 years, and III. articles should include original research describing colorectal cancer screening studies or tests proposed for future screening. An exception was made to point III in order to include meta-analyses and systematic reviews since they present the advantage of comparing many different studies all together to come to highly reliable conclusions. This was especially useful in some cases in which the amount of data recognized as possibly useful was excessively large. Furthermore, certain articles that were added after the initial research (as reported at the end of this section) did not respect the time restriction in point II.

The tests included in the guidelines have been excluded from our literature search as they are already in use and well described. An exception to this point was made in order to illustrate the Food and Drug Administration (FDA)-approved Cologuard^®^ MT-sDNA, seen as this test is still not widely utilised in common practice as other non-invasive tests of the same kind (e.g., FIT) are, and it is not yet recommended by the official European guidelines as a first line screening test. New imaging technologies were also excluded from our literature review. Though they prove to be promising in the field of CRC detection, due to their low cost-effectiveness—as reported by Thayalasekaran et al. [5]—no significant evidence supporting their use in large population-based screening programs was found.

These searches generated 555 articles, of which 93 titles were selected. Of these, 39 abstracts initially fulfilled the inclusion criteria and were further analyzed, and 12 were added as sources. As a result, a total of 51 articles were read, and of these, 10 were excluded. The tests cited in each of the remaining articles were also searched individually resulting in the addition of nine new papers. Finally, a total of 50 studies, describing 14 different CRC diagnostic tests, which are summed up in Table 1, were included.

### 2.1. Blood-Based Tests

#### 2.1.1. DNA Methylation

Methylation involves the covalent addition of a methyl group to a protein, DNA, or another molecule. DNA methylation plays an important role in the development of cancer by changing the gene expression. 

In particular, in cancer, DNA is generally hypomethylated and presents focal hypermethylation areas. These areas are often found in the promoter regions of tumor suppressor genes leading to their epigenetic silencing. Moreover, generalized hypomethylation in the gene bodies leads to genomic instability.

With regards to CRC, when comparing colorectal cancer cells with normal colorectal epithelial cells, it has been found that more than 10% of the protein-coding genes are differentially methylated [7,8].

#### 2.1.2. ctDNA Methylation—SEPT9 Methylation Assays

One of the genes for which methylation has been linked to CRC development is SEPT9 [9,10].

Various SEPT9 gene methylation assays have been developed based on the assumption that the risk of CRC development can be assessed by identifying the degree of DNA methylation of the promoter region of the SEPT9 gene in peripheral blood. The DNA that is searched in this blood-based test is called circulating tumor DNA (ctDNA), because, as the name suggests, cancer cells release it into the peripheral blood from necrotic and apoptotic processes during carcinogenesis [11]. Moreover, it is also actively released from exosomes [12].

At present, there is only one SEPT9 methylation assay kit already approved by the FDA as a valid alternative to CRC screening tests that have already been included in guidelines. This kit, called Epi proColon^®^ 2.0, is to be used on adults, age 50 or above, at average risk for CRC.

It uses a real-time polymerase chain reaction (PCR) with a fluorescent hydrolysis probe for the detection of specific methylation in the SEPT9 DNA target [11,13].

The study that led the FDA to approve this test was the PRESEPT study, which started in June 2008 and finished in April 2010 with the approval of Epi proColon^®^ 1.0 [14]. This study was performed in a screening setting of an average-risk population, ranging from 50–75 years old. The reported sensitivity was 48.2% (using a 1/2 algorithm). In 2011, a second-generation test (Epi proColon^®^ 2.0), characterized by enhanced diagnostic performance and technical advancements such as lower reagents and shorter turnaround time, was launched. The sensitivity increased to 68.2%, with a reduction in specificity to 80%, in a later study by Potter et al. who performed triplicate PCRs (1/3 algorithm) using samples from the same study [15]. In 2016, the FDA approved the Epi proColon^®^ 2.0, based on the data presented in the PRESEPT study with a 1/3 algorithm [11].

The necessary number of PCR tests to be performed in order to get a positive result is the basis on which the choice of which algorithm to employ is made.

The possible algorithms from which to choose from are the following: 1/3 algorithm, employed when one positive count out of three PCRs is needed; 1/2 algorithm, employed when one positive count out of two PCRs is needed; 2/3 algorithm, employed when two positive counts out of three PCRs are needed; and 1/1 algorithm, employed when one positive count out of one PCR is needed [11].

Out of the studies that met our initial criteria, four of them, conducted in China and Taiwan between 2016 and 2018, proved the efficacy of SEPT9 Methylation assays tests. Some of these also employ kits different from the Epi proColon^®^ 2.0. These are listed in Table S1 (Supplementary material) [16,17,18,19].

**Table 1:** Review summary.

	**CRCs vs. Controls**			**AAs vs. Controls**			
Guidelines Recommended Techniques *		**Sensitivity**	**Specificity**		**Sensitivity**		
Colonoscopy		0.95	0.86		0.75–0.95		
gFOBT		0.70	0.92		0.07–0.24		
FIT		0.74	0.96		0.08–0.24		
MT-sDNA		0.92	0.87		0.17–0.42		
**New approaches and perspectives**	**AUC range**	**Sensitivity range**	**Specificity range**	**AUC range**	**Sensitivity range**	**Specificity range**	**References**
**Blood based tests**							
mSEPT9	0.76–0.87	0.47–0.82	0.80–0.96	NA	0.09–0.59	NA	[16,17,18,19]
Methylated genes panels	0.86–0.97	0.74–0.97	0.72–0.97	0.94 [20]	0.89 [20]	0.86 [20]	[20,21,22,23]
Methylated genes in WBCs	0.72–0.98	0.30–0.90	0.96–0.98	NA	NA	NA	[24]
miRNA panels	0.68–0.96	0.65–0.89	0.26–0.93	0.91–0.95	0.94–0.95	0.85–0.90	[25,26,27,28,29,30,31]
piRNA panels	0.88–0.90	0.86–0.89	0.65–0.94	NA	NA	NA	[32,33,34]
Protein panels	0.75–0.99	0.56–0.99	0.80–0.99	0.60 [35]	0.80–0.90 [35]	0.22–0.32 [35]	[35,36,37,38,39,40]
Lipidic markers	0.93 [41]	0.85 [41]	0.89 [41]	NA	NA	NA	[41,42]
**Stool based tests**							
Tumor-M2-PK	0.71–0.92	0.63–1.00	0.40–1.00	NA	0.20 [43]	0.54 [43]	[43,44,45]
Gut microbial markers	0.72–0.84	0.56–0.69	0.77–0.81	NA	0.20–0.31 [46]	NA	[46,47,48,49]
Stool VOCs	0.76–0.82	0.27–0.95	0.58–0.95	NA	0.17–0.33 [50]	0.88–0.95 [50]	[50,51,52,53,54,55,56,57,58]
**Urine based tests**							
Urinary VOCs	0.67–0.98	0.63–1.00	0.42–0.95	0.54–0.61 [59]	NA	NA	[59,60,61,62,63]
Urinary ctDNA	0.96 [64]	0.73–0.91	0.82–0.85	NA	NA	NA	[64,65]
**Other:**							
Exhaled Breath VOCs	0.84–0.98	0.90–0.95	0.64–0.93	NA	NA	NA	[66,67,68]
Saliva miRNAs	NA	0.97	0.91	NA	NA	NA	[28]

* Chyke Doubeni, Tests for screening for colorectal cancer. In: UpToDate, Post, TW (Ed), UpToDate, Waltham, MA, 2020. [69] Abbreviations: CRC = colorectal cancer; AAs = advanced adenomas; gFOBT = guaiac fecal occult blood test; FIT = Fecal immunochemical test; Mt-sDNA = Multi-target Stool DNA Test; WBCs = white blood cells; miRNA = microRNA; piRNA = Piwi-interacting RNA; Tumor-M2-PK = dimeric pyruvate kinase isoenzyme M2; VOCs = Volatile organic compounds; ctDNA = ctDNA = Circulating tumor DNA.

These studies demonstrated a variable sensitivity (ranging between 47–82%) and specificity (ranging between 81–95.9%). They also showed a good sensitivity range for early stages disease (stage I and II) around approximately 60% for stage I and 70% for stage II.

In general, these results appear to be comparable to the ones obtained by the PRESEPT study using Epi proColon^®^ 2.0 and confirm the feasibility of SEPT9 Methylation assays test as useful options to screen CRC patients.

In Table 2, the focus lays on the efficacy of SEPT9 when combined with other commonly used tests such as FIT or carcinoembryonic antigen (CEA).

While the PRESEPT study investigated the efficacy of Epi proColon^®^ in a population-based mass screening, the other mentioned authors studied SEPT9 methylation assays in an opportunistic screening setting. This type of screening occurs when potential subjects willingly undergo examinations or tests because of illness or discomfort or, even if without symptoms or complaints, request to be screened early for CRC outside of a current screening program. This model presents the disadvantage of only taking into consideration patients who seek medical care, leaving others out of the program. Nevertheless, it still represents a crucial diagnostic and preventive tool.

All these recent studies were conducted in Asian populations, causing the risk of producing misleading results when referring to other ethnicities. Song et al. [64] addressed this issue comparing their findings observed on a Chinese population to the results obtained by Potter et al. [15] on Caucasian and African American populations in 2014.

The false positive rate of mSEPT9 assay was similar among Chinese and Caucasian populations in every age group, whereas the African American group between 50–59 years of age exhibited a significantly higher false positive rate than the other two ethnicities.

Also, the older population, over 60 years of age, exhibited higher false positive rates than the younger population in Chinese and Caucasian populations. Furthermore, the overall detection rate showed a significant difference between the study conducted by Song, where it was 83.8%, and Potter’s one on African American and Caucasian populations, where it was of the 68.2%.

However, even though a 1/3 algorithm was employed in both studies, the considered kits presented key differences: the PRESEPT study adopted the Epi proColon^®^ 1.0 assay while Song’s study adopted the Epi proColon^®^ 2.0 assay. For this reason, the author concluded that these observations alone are not enough to affirm whether there is a difference between Chinese and other ethnicities. Therefore, this topic needs further investigation [17].

In conclusion, different studies demonstrated a variable sensitivity of the FOBT tests, ranging from 33–79% [70,71]. The sensitivity of Epi proColon^®^ 2.0 and the commercialized mSEPT9 assay did not differ from the most widely used Fecal immunochemical tests (FIT), which has a sensitivity up to 79% at 94% specificity [70,71,72]. When taking into consideration the fact that FOBT tests have a low compliance in terms of screening uptake [73] and many factors can lead to a false-positive result such as inflammation, infections, ulcers, and hemorrhoids, the mSEPT9 assay, which is not affected by those factors, could be considered superior, in terms of detection rate, to the fecal test. However, because of the higher cost-effectiveness, FIT remains the first choice among diagnostic tests for CRC screening.

It is also notable that different studies found that mSEPT9 assay sensitivity was further enhanced when it was combined with carcinoembryonic antigen (CEA) or FIT. Consequentially, a combined MS-9 DNA blood test and FIT/CEA may help to achieve a higher detection rate of CRC and may represent a valid option for screening.

#### 2.1.3. DNA Methylation in White Blood Cells (WBCs)

As mentioned before, DNA methylation can be found in circulating cells in peripheral blood and some evidences show that these cells can either be circulating tumor cells or white blood cells (WBCs) [74].

A 2019 study by Boonsongsermt et al. [24] in Bangkok investigated the changes in DNA methylation of peripheral blood mononuclear cells (PBMCs) among normal individuals and CRC cases using a methylation microarray.

The cohort included 32 CRC patients and 57 normal controls, both investigated directly by colonoscopy. PLOD1 and MMP9 were selected to assess the DNA methylation of the WBCs from CRC patients using real-time methylation-specific PCR. MMP9 showed high diagnostic efficacy with 90.63% sensitivity, 96.49% specificity, and high Positive Predictive Value (PPV) (93.33%), and Negative Predictive Value (NPV) (93.22%). On the other hand, the PLOD1 methylation test showed high specificity (97.92%) but low sensitivity (30%). Moreover, neither of these methylation changes were found to correlate with tumor grade or stage. Although this study was limited by its small sample size, it still paves the way for future screening setting studies.

### 2.2. Panels of Methylated Genes

SEPT9 only represents one of the many genes associated with CRC to present methylation alterations. These methylation patterns are usually studied and analyzed through specific techniques among which we remember the methylation affinity-based isolation, the methylation-specific restriction enzyme digestion, and the chemical modification of cytosine with sodium bisulphite conversion [20,75,76].

Four studies, exploring the feasibility of panels of methylated genes as CRC screening tests, are summarized in Table S2 (Supplementary material) [20,21,22,23].

These studies demonstrated a varying sensitivity (ranging between 73.9–96.6%) and specificity (ranging between 72.5–97.3%) for different gene panels. Notably, the panel studied by Bartak et al. [20], constituted by SFRP1, SFRP2, SDC2, and PRIMA1 gene promoters, showed 89.2% sensitivity and 86.5% specificity (AUC: 0.937, 95% CI: 0.885 to 0.989) for adenoma detection, which represents a crucial feature of any good CRC screening test.

Another significant result was achieved by Freitas et al. [22], using a panel including MGMT, RASSF1A, and SEPT9, that was able to identify tumors at any disease stage with similar efficiency (sensitivity of 100%, 94.2%, 95.9% for stage I/II, stage III and stage IV, respectively) with PPV 91.5% (AUC = 0.97).

Because the disease prevalence in those screening-like cohorts is low, in general, large sample sizes are essential and this poses a significant logistical and economic barrier to the correct assessment of a reliable model [75]. Notably in some of these studies, despite the fact that a very large screening population was recruited, the final sample was small, constituting a limitation to the statistical reliability of these results. Nonetheless, some of these panels revealed to have very high detection rates and may be worth further exploration in larger screening setting studies.

#### 2.2.1. RNA

As mentioned before, methylation plays a variety of roles in cancer, changing the regulation of gene expression. The (epi) genetic alterations that drive the transformation from normal colon epithelium into adenocarcinoma can affect noncoding RNAs and mRNAs as well [77] and therefore, investigating them may represent a valuable option for the early diagnosis of CRC.

#### 2.2.2. miRNA

MicroRNAs are small and non-protein-coding RNAs, molecules of 21–25 nucleotides long, that regulate gene expression and exhibit important regulatory functions related to cell differentiation, development, and growth. There is evidence that the levels of some miRNAs are altered in cancers such as CRC and that miRNAs regulate the cancer-promoting RAS gene [78]. This is why many authors and studies have focused on finding miRNAs useful in the early detection of CRC.

For example, a meta-analyses incorporating 103 studies with a total of 3124 CRC patients and 2579 healthy individuals, performed by Yan et al. [25] in 2016, found that miRNAs have a good performance in detecting CRC with 76.9% sensitivity (95% CI 0.733–0.802), 80.6% specificity (95% CI 0.781–0.829), and AUC 0.857. With subgroup analysis and meta-regression, they also proved how multiple miRNAs seem to be more favorable than single miRNA (sensitivity 0.853 > 0.718, specificity 0.860 > 0.772, AUC: 0.918 > 0.813,). They also noticed how comparing samples of plasma, blood, tissue, and feces, miRNAs obtained from serum samples were more powerful for detecting CRC, particularly in Asians.

Another recent meta-analyses, published in 2017 by Carter et al. [26] based on a search result of 34 articles, found a total of 31 miRNAs to be either upregulated (*n* = 17) or downregulated (*n* = 14) in CRC cases as compared to controls. In this analysis, 14 studies identified panels of dysregulated miRNAs and the highest AUC, 0.943, was identified using a panel of four miRNAs (miR-29a, -92a, -601, -760), with 83.3% sensitivity and 93.1% specificity (Wang et al., 2012 [79]). They found that the overall sensitivity and specificity of 28 individual miRNAs in the diagnosis of CRC were both 76% (95% CI 0.72–0.80), indicating good discriminative ability of miRNAs as biomarkers for CRC.

In the vast amount of studies investigating miRNAs as CRC biomarkers, we further selected five up-and-coming studies from the last 5 years, which are listed in Table S3 (Supplementary material) [27,28,29,30,31].

Noticeably, in the study conducted by Sazanov et al. [28], microRNA-21 was found to be expressed in saliva as well and showed higher diagnostic efficacy than miR-21 expression in the plasma, with a sensitivity of 97% and specificity of 91%, at confidence interval of reference values 0.65–2.49 (*p* > 0.95). Even with the small sample size, these results are important, considering that a saliva-based test is a non-invasive and cheap procedure, which is perfectly suitable in a screening setting.

As suggested by several authors, miRNA definitively represents a potential specific biomarker for CRC detection [25,26,27,28,29,30,31]. However, there are currently no relevant diagnostic products widely available for clinical use, meaning that additional research is warranted to implement these markers for clinical use in a screening setting.

#### 2.2.3. piRNA

piRNAs or Piwi-Interacting RNAs represent a novel class of small non-coding RNA molecules expressed in animal cells. They form RNA–protein complexes with a subset of Argonaute proteins of the piwi-family, and these complexes are mostly involved in the epigenetic and post-transcriptional silencing of transposable elements and other spurious or repeat-derived transcripts [80]. Recent studies have shown that the expression of piRNAs is frequently deregulated in different types of cancers including CRC [81]. Because of their small size, high stability, and ease of detection, piRNAs represent a strong diagnostic and predictive biomarker [82] with high potential as CRC screening tools.

Three studies describing piRNAs as CRC screening tests were listed in Table S4 (Supplementary material) [32,33,34].

Particularly, the panel, described by Wang et al. [34], composed of piR-020619 and piR-020450 showed up to 88.75% sensitivity and 93.75% specificity for the overall CRC detection and up to 76.79% sensitivity and 90.94% specificity (AUC: 0.839) for CRC stage I–II. Notably, this piRNA panel remained a strong predictor of CRC regardless of the subgroups of the patients taken into consideration in the training and validation cohorts. Moreover, the expression levels of these piRNAs in the sera of lung, breast, and gastric cancer patients were similar to those of normal controls, suggesting that piR-020619 and piR-020450 could serve as CRC-specific biomarkers.

Another significant result was obtained by Mai et al. [33] that investigated piR-54265, for which pre-diagnostic serum levels were significantly associated with future CRC diagnosis, (ORs of 7.23, 2.80, 2.45, and 1.24 for those whose CRC was diagnosed within 1, 2, 3, and >3 years, respectively). piR-54265 showed 83% sensitivity and 65.1% specificity [AUC: 0862 (95% CI, 0.827–0.891), *p* < 0.001] for CRC stage I–II.

### 2.3. Protein Panels

The number of protein molecules indicated by the literature as possible CRC markers in blood is wide. However, only two are currently the main blood-based biomarkers available to detect CRC patients: carcinoembryonic antigen (CEA) and carbohydrate antigen 19-9 (CA19-9).

CEA is a high-molecular-weight glycoprotein and it is expressed in embryonic tissue as well as colorectal malignancies. CEA is particularly useful when used as a prognostic factor (poor prognostic factor for resectable CRC, cancer progression and recurrence after surgery) but when used as a tool for early detection in a screening setting its sensitivity is low because its levels are strictly related to the tumor stage. Moreover, CEA is not specific for CRC, but a higher level can be caused by liver disease, pancreatitis, Inflammatory Bowel Disease (IBD), and other malignancies. On the other hand, CA19-9 antigen is even less sensitive and specific for CRC, while it represents a highly reliable marker for the detection of pancreatic and biliary malignancies [83,84].

These observations point to the need for new molecules with higher reliability in the early detection of colorectal cancer. As previously mentioned, the number of markers recognized as possibly useful for this task is large; for this reason, the following section will also include evidences collected through systematic reviews. These works present the advantage of comparing many different studies concomitantly to come to highly reliable conclusions.

Particularly, Bhardwaj et al. [36] published the PRISMA systematic review in 2017 based on 36 studies, which unfortunately were performed in a clinical and not in a screening setting, ranging from 23–512 cases of CRC and a number of proteins included in the signature ranging from 3–13. What they found is that, among the 21 studies that performed some form of validation, the best diagnostic performance was reported by Zhang et al. [37] for a panel including CA199, CA242, CA125, CA153, and CEA, which showed to have 94% sensitivity, 98% specificity, and AUC 0.988. They also investigated studies that did not use any form of validation, and among these the best diagnostic performance was found for a combination of inflammatory markers (IL-8, MMP-2, TNF-a) found by Pengjun et al. [38], which presents 96% sensitivity, 99% specificity, and AUC 0.996.

The same author performed a two-stage study in 2020 [39], aiming to measure 275 protein markers. They used the proximity extension assay (PEA) in plasma samples with a discovery set, which included 98 newly-diagnosed CRC patients and 100 age-and-gender-matched healthy controls screened with colonoscopy. Moreover, an independent external validation set was also used. This set included 56 CRC patients and 102 healthy controls, recruited from a pool of 945 participants of a true screening study. They found a 12-markers algorithm (including AREG, CEA, GZMB, ITGAV, KRTI9, MCP1, PON3, TR, MASP1, RARRES2, S100A4, and TRAP), particularly promising in the detection of early stage CRC, that in the validation set showed 61% sensitivity and 80% specificity, with an AUC = 0.75 (95% CI 0.67–0.84), comparable with the above-mentioned Epi proColon 2.0.

Moreover, the potential of AREG as an excellent diagnostic tool was also studied by Chen et al. in the BLITZ study [35]. In this two-stage design study in China with a discovery set and a validation set including 7197 participants, they studied a panel made up of four protein markers (GDF-15, AREG, FasL, Flt3L) + serum level of TP53 autoantibody. Their results showed 66.7% sensitivity and 80% specificity for the panel not including serum TP53 autoantibody, and 56.4% sensitivity and 90% specificity for the panel including it. These values are comparable to the diagnostic performance of Epi proColon^®^ 2.0.

However, the panel presented limited diagnostic efficacy in detecting advanced adenomas, with an AUC of 0.60 (95% CI, 0.52–0.69). Furthermore, despite the very large screening population recruited, the sample size of CRC cases included in the validation set was small, reflecting the very low prevalence of CRC in a true screening population.

Finally, Loktionov et al. [40] completed another review in 2020 based on various works of research investigating CRC associated proteins. Even if most of the studies produced modest results, the authors pointed out some promising molecules to be used in a screening setting that are worth a mention: CA11-19, TFF3 (Nikolau et al., 2018 [85]), Cyr61 (Song Y. et al., 2017 [86]), and B6-integrin (Bengs et al. 2019 [87]). These molecules are to be intended as single protein markers also because, as the author noted, panels of proteins are more technically complex and expensive to realize. Despite this, the review still highlights the results obtained by Jiang et al. (2014) [88] with a panel composed of lectins DC-SIGN and DC-SIGNR that showed a sensitivity of 98.7% and a specificity of 94.8%. Moreover, this panel has also been pointed out by the Nikolau et al.’s [85] review (mentioned above) which, in 2018, compared 51 studies and found CA11-19, DC-SING and DC-SIGNR, and IL8 to be interesting diagnostic biomarkers.

When looking at this data, it is evident how further research conducted in larger screening setting studies could pave the way for new efficient tests available for the clinical routine. At this moment, the current evidence is still insufficient when comparing protein biomarkers with the cost-effective and already widespread FIT.

### 2.4. Combination of Protein and Genes Panels

Omitted from our review because it is a non-CRC specific cancer screening test, but still described because of its relevance as feasible future cancer screening tool, is CancerSEEK.

In 2018, Cohen et al. developed this blood test that detects multiple types of cancer, including CRC, by combining the detection of circulating free DNA (cfDNA) and protein biomarkers (including CA-125 and CEA) that are released by tumors [89,90].

This test works with an algorithm that weighs the protein and DNA data collected from the blood in order to detect patients who are likely to have a tumor.

Preliminary performance of the test was evaluated in a set of 1005 individuals with known cancers who were compared with 812 healthy controls.

Tumor type and location influenced the accuracy of the prediction: the highest accuracy was achieved for colorectal cancer. For lung cancer it was the lowest. In particular, specificity of the test was over 99% in eight cancer types: ovarian, liver, stomach, pancreatic, esophageal, colorectal, breast, and lung. Sensitivity ranged from approximately 98% in ovarian and liver cancer to 33% in breast cancer, with a sensitivity of about 70% for the remaining cancers. Moreover, the tissue of origin was correctly identified in approximately 80% of patients.

Although the false-positive rate was low in the trial, it would be expected to be higher in the real-world setting when the test is applied to a healthy population without known diagnosis of cancer. In fact, the authors weighted the results for the actual incidence in the United States and estimated a sensitivity of 55% among the eight cancer types.

In conclusion, the authors stated that multi-analytic tests, such as this one, are not meant to replace other non-blood-based screening tests—such as those for colorectal cancer—but are meant to provide additional information that could help identify and diagnose patients who are at higher risk of having a malignancy [90,91].

### 2.5. Lipidic Markers

In recent years, there has been a growing interest in lipids as potential biomarkers in numerous clinical conditions, and lipidomic studies represent a new important tool in monitoring CRC patients. In clinical practice, lipid status is estimated based on serum concentrations of total cholesterol, HDL, LDL, and triacylglycerols. However, other currently available techniques, for instance mass spectrometry, may provide a more detailed insight into the structure and function of these molecules as well as into the lipid profile of CRC patients [92].

Often, these lipid alternations in patients with CRC have been studied and investigated as prognostic factors or markers for late stage disease but modest are the results obtained when looking at these molecules as screening test tools. China has been the setting for a few promising studies focused on free fatty acids (FFAs) and their products as potential biomarkers to screen CRC patients: they took place in Beijing and Zhanjiang (Guangdong), respectively.

Zhang Y. [41] performed a two-stage study based on a training set, which included 59 CRC patients and 69 healthy controls. Moreover, a validation set was also used. This set included 80 CRC patients, 55 Benign Colorectal Diseases (BCD) patients, and 116 healthy controls in which the levels of Serum Unsaturated Free Fatty Acids were evaluated using Mann–Whitney U tests to compare patients and controls. The results showed excellent diagnostic performance for a pool of four Unsaturated FFAs (C16:1, C18:2, C20:4, C22:6) with 84.6% sensitivity, 89.4% specificity, and AUC 0.926.

Along this direction, Zhang L. [42] investigated Poly-unsaturated fatty acid (PUFA) metabolites, inflammatory mediators that can affect progression and treatment of cancer. Ultra-high-performance liquid chromatography tandem mass spectrometry (UPLC-MS/MS) was used to assess their levels in a cohort of 25 CRC patients and 10 healthy controls. Of the 158 PUFA and metabolites studied, they found the following abnormal changes in CRC patients: of 2, 3- dinor-8-iso-PGF2α, 19-HETE, and 12-keto-LTB4 from arachidonic acid and significant lower levels of 9-HODE and 13-HODE from linoleic acid. So far, the results obtained by studying these molecules are modest, but this data could be taken into consideration for the set-up of larger screening setting studies that could lead to the implementation of lipidomic profile as an important tool for CRC detection.

### 2.6. Stool-Based Tests

#### 2.6.1. Multitarget Stool DNA (MT-sDNA) Test

As mentioned before, the American Cancer Society guidelines already recommend the Multitarget Stool DNA test as a feasible option to screen the average-risk population, for this reason it was omitted from our literature search. Nonetheless, we decided to dedicate a section to the FDA-approved MT-sDNA test, called Cologuard^®^, seing as this test is still not widely utilized in common practice as other non-invasive tests of the same kind (e.g., FIT) and it is not yet recommended by the official European guidelines as a first line screening test.

Cologuard^®^ is a molecular assay for aberrantly methylated BMP3 and NDRG4 promoter regions, mutant KRAS, and β-actin, which is used as a reference gene for DNA quantity, combined with an immunochemical assay for human hemoglobin.

The study that granted the FDA approval was a cross-sectional study conducted by Imperiale et al. in 90 different areas throughout the United States and Canada, from June 2011 through November 2012. It enrolled 9989 asymptomatic individuals between the ages of 50–84 years who were considered to be at average risk for colorectal cancer and who were scheduled to undergo screening colonoscopy. It compared the MT-sDNA test with FIT only and the results were generated with the use of a logistic-regression algorithm, with a positive score threshold of 183 or more considered to be positive.

DNA testing showed 92.3% sensitivity for CRC and 42.4% for advanced adenomas (AAs).

FIT showed 73.8% sensitivity for CRC (*p* = 0.002) and 23.8% for AAs (*p* < 0.001).

Moreover, polyps with high-grade dysplasia and serrated sessile polyps measuring 1 cm or more were detected with a rate of respectively 69.2% and 42.4%. However, DNA testing showed lower specificity than FIT: 86.6% compared to FIT’s 94.9% specificity among patients with nonadvanced or negative findings (*p* < 0.001) and 89.8% compared to FIT’s 96.4%, among those with a negative colonoscopy result (*p* < 0.001).

These results show that the MT-sDNA test, in asymptomatic persons at average risk for colorectal cancer, detects significantly more cancers than FIT. Therefore, as the authors suggested, being a noninvasive test with a high single-application sensitivity for curable-stage cancer, Cologuard^®^ may provide a suitable option for persons who prefer noninvasive testing, although with lower specificity [93].

#### 2.6.2. Tumor M2-PK

Pyruvate kinase isoenzyme type M2 (M2-PK) is a pyruvate kinase isoform present in differentiated tissues, such as lung tissue, fat tissue, the retina, pancreatic islets, and highly proliferating cells like fibroblasts, lymphocytes, embryonic cells, adult stem cells, and colonocytes, and it is up-regulated in many types of tumor [94,95]. Usually pyruvate kinase isoenzymes in their active form in healthy tissues are tetramers. Tumor cells instead contain high levels of almost-inactive dimeric M2-PK, which, for this reason, has been named “Tumor M2-PK”. Signal metabolites in tumor cells influence the ratio between M2-PK tetramer and dimer. This regulation is crucial because M2-PK plays a role in cell cycle progression and supports anabolism and tumor growth in several contexts [94,96]. Concerning colorectal cancer, researchers found that the human papillomavirus (HPV)-16 E7 protein, which concurs with the k-RAS gene in cell transformation, interacts with PKM2, inducing and stabilizing the tumor form of M2-PK [97].

Because colonocytes are shed into the gut lumen, t-M2-PK can be detected in stool samples by an enzyme-linked immunosorbent assay (ELISA), making fecal determination of this isoenzyme a useful test for early detection of CRC. Its evaluation gave very encouraging results in pilot studies in 2004 and 2006 [97,98,99], paving the way for more studies. Three, among the papers we initially selected, described t-M2-PK as a promising tool for CRC detection and are therefore described in Table S5 (Supplementary material) [43,44,45].

Among these, Che Alhadi et al. [43] compared the M2-PK test with gFOBT and obtained promising results: the sensitivity of the M2-PK test was higher than gFOBT (100.0% vs. 64.7%), however, with a lower specificity (72.5% vs. 88.2%). [AUC = 0.868 (95% CI: 0.794–0.941; *p* < 0.005)].

#### 2.6.3. Gut Microbiota as CRC Screening Tool

The gut is the home of a large microbial community containing bacteria, fungi, and viruses for a total number that may reach 100 trillion. This flora live in a mutualistic relationship with his host, filling many roles: It enhances the epithelial defense against pathogens, accelerates the maturity of the immune system, protects the local homeostasis, and also shows endocrine functions. Particularly, human gut microorganisms ferment dietary fiber into short-chain fatty acids, which are subsequently absorbed, promote the synthesis of vitamin B and vitamin K, and are involved in the metabolization of bile acids, sterols, and xenobiotics. These aspects clearly show how the dysregulation of the gut microbiota can affect the immune response and play a role in the development of inflammatory and autoimmune conditions [100,101,102,103].

According to studies on twins and relatives, it is estimated that the heritability of CRC is only 12–35% and genetic predisposition syndromes for CRC only account for a minority of CRC cases [104]. This relatively low level of heritability of CRC reflects the importance of environmental factors and among them the role of microorganisms has been increasingly recognized over the years.

Findings show that the gut microbiota of CRC patients is different compared with the gut microbiota of healthy individuals, containing higher richness of species, a lower quantity of potentially protective bacteria and increased presence of procarcinogenic bacteria such as Bacteroides, Escherichia, Fusobacterium, Peptostreptococci, and Porphyromonas [47,104,105].

For this reason, in Table S6 (Supplementary material) [46,47,48,49], we described four recent studies aiming to identify CRC patients using PCR to analyze bacteria in the stool sample of individuals undergoing colonoscopy or FIT.

Among them, Elkof et al. [46] had the most promising results: F. nucleatum identified CRC patients with 69.2% sensitivity and 76.9% specificity.

These results are consistent with numerous prior observations on the pathogenic mechanisms behind the proportional association between Fusobacterium and CRC. In particular, it has been found that Fusobacterium is able to alter the tumor microenvironment by myeloid-derived suppressor cells, tumor-associated macrophages and neutrophils recruitment, and local immune suppression. Moreover, it is responsible for the activation of the E-cadherin/β-catenin signaling pathway and through epigenetic changes such as microsatellite instability and hypermethylation can produce malignant transformation of epithelial cells [106].

### 2.7. Volatile Organic Compounds (VOCs)

#### 2.7.1. Stool VOCs

In the recent past, there has been an increasing interest in the human volatilome as a potentially non-invasive diagnostic biomarker in clinical medical practice. VOCs can be defined as the spectrum of volatile organic chemicals originating from (patho)physiological metabolic processes in the human body and detectable in a large range of secretions. A 2014 review by de Lacy Costello et al. reported 1765 volatile compounds to appear in exhaled breath (*n* = 872), saliva (*n* = 359), blood (*n* = 154), milk (*n* = 256), skin secretions (*n* = 532) urine (*n* = 279), and feces (*n* = 381) in apparently healthy individuals [107,108].

Lately, different studies have evaluated the usability of VOCs present in the headspace of feces as diagnostic tools for gastrointestinal diseases. Particularly in those diseases in which microbiota alterations occur such as colorectal carcinoma in which, as mentioned above in this review, microbial agents are considered to play a significant etiological role.

Currently, VOC detection techniques can be divided into two different categories: chemical analytical techniques for the quantitative and qualitative detection of individual VOCs and pattern-based techniques, using electronic devices containing an array of different VOC sensors that compare the total set of gases with a pattern recognition algorithm. On one hand, Gas chromatography-mass spectrometry (GC-MS), which is a chemical analytical technique, is considered the ongoing gold standard in VOC detection [52], but on the other hand, VOC sensors are getting more and more relevance because they are inexpensive, often portable, and easy to use [109]. This is why findings such as the ones listed in Table S7 (Supplementary material) could represent the next frontier for large population-based screening programs [50,51,52,53,54,55,56,57,58].

Among them, SCENTA1^®^ [58], that works using a pattern-recognition technology and was recently patented by Zonta et al. [57], showed remarkable sensitivity and specificity (95%). The authors suggest that the addition of a technology that relies on a gaseous marker, totally different from occult blood, as a complementary test could drastically reduce false positive rates. However, since the sample size was small, further studies will be necessary to validate these findings.

#### 2.7.2. Breath VOCs

For the convenience of the acquisition of samples, VOCs are gaining importance in cancer screening studies. These studies use stool and different secretions such as exhaled breath, aiming to find smart sensory tests that are able to facilitate the diagnosis and increase screening adherence in the population. For example, Wang et al. [66], collected exhaled breath of 20 CRC patients and 20 healthy controls in their study in China, in 2014. They used solid-phase microextraction methods including gas chromatography/mass spectrometry (SPME-GC/MS) to assess the participants’ VOC pattern. The mean age of the patients in the cancer group was 58.1 years, with a standard deviation of 14.2 years. The team used the statistical methods of principal component analysis (PCA) and partial least squares discriminant analysis (PLS-DA) to process the final data. Significant differences were found in VOCs in the exhalations of CRC patients compared to the VOCs in the exhalations of healthy controls. In fact, CRC patients presented higher levels of cyclohexanone, 2,2-dimethyldecane, dodecane, 4-ethyl-1-octyn-3-ol, ethylaniline, cyclooctylmethanol, trans-2-dodecen-1-ol, and 3-hydroxy-2,4,4-trimethylpentyl 2-methylpropanoate, and significantly lower levels of 6-t-butyl-2,2,9,9-tetramethyl-3,5-decadien-7-yne (*p*  <  0.05).

More recently, in a case control study in Italy, Altomare et al. [67] studied the breath print of 83 patients with colorectal cancer and 90 non-cancer controls collected using Gas chromatography–mass spectrometry. They used ROC curve analysis to discriminate the ability of each VOC in detecting colorectal cancer and finally cross-validated the results by the leave-one-out method and applying stepwise logistic regression analysis. Fourteen VOCs (tetradecane, ethyl- benzene, methylbenzene, acetic acid, 5,9-undecadien- 2-one, 6,10-dimethyl (E), decane, benzaldehyde, benzoic acid, 1,3 bis(1-metiletenil) benzene, decanal, unidenti- fied compound T22_75, dodecane, 2-ethyl-1-hexanol and ethanone, 1[4-(1-methylethenyl)phenyl]) were found to have significant discriminatory ability in detecting patients with colorectal cancer. The model presented an AUC of 0.979 and further cross-validation of the model resulted in a true predictive value for colorectal cancer of 93% overall, 90% sensitivity, and 93% specificity. Moreover, the reliability of the breath analysis was maintained no matter the cancer stage with 86% sensitivity and 94% specificity for early stage disease.

Also, the above-mentioned pattern-based techniques are being explored in breath VOCs detection. We see this in Van Keulens’ et al. 2019 multicentered study in the Netherlands [68]. Their study was carried out on adult colonoscopy patients and evaluated exhaled volatile organic compounds using an electronic nose. This device, named Aeonose^®^ (The eNose Company, Zutphen, Netherlands), is a portable, battery-powered device, that contains three metal-oxide sensors with different material properties that create a patient “breathprint”. Inhaled air is also filtered to prevent contamination of the e-nose by environmental VOCs bacteria or viruses and the analysis takes 15 min of which the patient breathes into the device for 5.

In the study, 511 breath samples were collected with 70 CRC patients and 125 heathy controls. Training models for CRC and AAs had an AUC of 0.76 and 0.71 and blind validation resulted in an AUC of 0.74 and 0.61 respectively. Ultimately the final models that the authors found for CRC and AAs showed an AUC of 0.84 (sensitivity 95% and specificity 64%) and 0.73 (sensitivity and specificity 79% and 59% respectively).

From these results, it can be concluded that analysis of breath VOCs could represent an effective and convenient screening method for the disease, particularly when a device is capable of providing a binary answer (cancer/no cancer) to eventually direct to further work-out.

### 2.8. Urinary Tests

#### 2.8.1. Urinary VOCs

Over the years, the study of urinary volatilome has also been gaining importance. There are two methods currently available to differentiate between healthy controls and CRC patients: mass spectrometry (MS) and nuclear magnetic resonance spectroscopy (NMR) [110]. The method that is used more often is currently Ion Mobility Mass Spectroscopy (FAIMS), which is based on physical properties rather than chemical properties [111]. Particularly, it uses the differences in the electric field dependence of ionized chemical mobilities to separate chemical components. One of its advantages is that, unlike other similar analytical techniques, it can work at atmospheric pressure and room temperature [111].

#### 2.8.2. Field Asymmetric Ion Mobility Mass Spectroscopy (FAIMS)-Based Studies

In 2014, in the UK Arasaradnam et al. [60] investigated the VOC signature of 133 subjects (83 CRC patients and 50 healthy controls), with a mean age of the CRC patients of 60 years (standard deviation of 17 years), and 64% of males. Simultaneously with CRC diagnosis, urine was collected and headspace analysis was conducted using FAIMS. Fisher Discriminant Analysis was used to process the data, which demonstrated that the VOC profiles of CRC patients differed from the healthy controls with 88% sensitivity and 60% specificity. This result is lower compared to the gold standard colonoscopy, but it is comparable with current fecal stool testing including the gFOBT and FIT.

In 2018, Widlak et al. [61] conducted a large single-center, prospective, and blinded study on a subset of 562 patients with matching urine and stool samples (FIT and faecal calprotectin) who were included for final statistical analysis from an initial population of 1850 patients meeting criteria for inclusion. They used a commercial gas analysis instrument based on ion mobility spectroscopy (FAIMS) to analyze VOCs emanating from the urine samples. The results showed that the sensitivity and specificity for CRC using FIT was 80% and 93%, respectively, and for urinary VOCs it was 63% (95% CI 0.46–0.79) and 63% (95% CI 0.59–0.67), respectively. Notably, for CRC patients who were FIT-negative (false negatives), the addition of urinary VOCs to FIT resulted in a sensitivity of 97% (95% CI 0.90–1.0) and specificity of 72% (95% CI 0.68–0.76). The authors concluded that, when applied to a FIT-negative group, urinary VOCs improve CRC detection and can be considered a promising second-stage test to complement FIT in the detection of CRC.

In another study settled in the UK during 2019, Mozdiak et al. [59] analyzed the urine of 163 FOBT+ patients from a screening population using field asymmetric ion mobility spectrometry (FAIMS) and gas chromatography coupled with ion mobility spectrometry (GC–IMS). The collected data was analysed using a machine learning algorithm and the results showed a high test accuracy for differentiating CRC from control with a high degree of separation. Using GC–IMS, AUC was 0.82 (0.67–0.97) with sensitivity 80% (95% CI 0.44–0.97) and specificity 83% (95% CI 0.63–0.95). Using FAIMS, AUC was 0.98 (0.93–1) with 100% sensitivity (0.74–1) and 92% specificity (0.62–1). However, even though the separation of CRC patients from the normal controls was high, when CRC cases were grouped with adenomas, the accuracy dropped significantly (AUC range 0.83–0.92) and the independent grouping of adenoma and controls was poor with AUC range 0.54–0.61, using both modalities.

When looking at these results, we should notice some limitations: Current studies are limited to case control and cohort studies, not taking into consideration a real screening setting. Also, due to their complex interaction in the gut, external factors such as diet and medications play a role in VOCs profiling [50] and they are often not deeply analysed before proceeding into the CRC-control differentiation. Nonetheless, these studies highlight the potential of FAIMS in analyzing Urinary VOCs for CRC detection.

#### 2.8.3. Urine Nuclear Magnetic Resonance (NMR)-Based Studies

In 2016, Deng et al. [62] conducted a prospective study in Shanghai where they evaluated a novel urine-based metabolomic diagnostic test for colonic adenoma detection (PolypDx™) on 1000 Chinese patients undergoing a colonoscopy. This test was originally developed and validated on a Canadian Cohort [112].

Particularly, the PolypDx™ prediction algorithm utilizes concentrations of three key metabolites in urine sample, which is determined by one-dimensional nuclear magnetic resonance (NMR) analysis and participant clinical features (age, sex, and smoking history) to compute prediction results as positive (adenoma detected) or negative (adenoma not-detected). They calculated an AUC of 0.717 and a sensitivity and specificity of 82.6% and 42.4% respectively. If we compare these results to the fecal-based tests, the specificity is lower. However, we should consider that these fecal tests were designed to detect colorectal cancer, and not all polyps. MTI’s urine-based test is instead designed to detect adenomatous polyps which, as the authors notice, makes it appropriate to serve as a population-based screening tool for CRC.

An analysis of effectiveness and cost-effectiveness of PolypDx™ test every 2 years, in comparison to screening strategies such as colonoscopy every 10 years, annual gFOBT, and annual FIT, was performed in 2018 by Barichello et al. [113]. They found that despite the higher cost (incremental cost-effectiveness ratio (ICER) at $46,783 vs. $51,616, $29,568, and $31,008 respectively), the metabolomics-based urine screening strategy compared with the other techniques was the most effective method. It was found to be correlated with a reduction in mortality by 41% and a gain of 0.13 life-years per person (vs. reduction by 25% and 0.08 life-years gained, reduction by 15% and 0.04 life-years gained, and reduction by 36% and 0.11 life-years gained, respectively) and, in conclusion, a cost-effective strategy.

In 2019, another study by Kim et al. [63] explored the potential of urine NMR metabolomics. Urine samples from 92 patients with colorectal neoplasia and 156 healthy controls were collected and analyzed. The team, using the Orthogonal Projections to Latent Structures Discriminant Analysis (OPLS-DA) model, found a metabolomics profile consisting of taurine, alanine, and 3-aminoisobutyrate to be a good discriminator for CRC patients with an AUC of 0.823, 0.783, and 0.842 respectively. The sensitivity and specificity for diagnosing pre-invasive colorectal neoplasia was 96.2% and 95%, respectively, revealing the urine-NMR metabolomics to be a high potential screening tool for accurate diagnosis of pre-invasive CRC.

#### 2.8.4. Urinary Circulating Tumor DNA (ctDNA)

Circulating tumor DNA molecules can be found in various body fluids, and CRC, notoriously, has a high tumor cell loss factor into the peripheral blood from necrotic and apoptotic cancer cells that occurs during carcinogenesis. While ctDNA has been extensively studied in serum, only a couple of studies focus on their presence in different bodily fluids to distinguish between CRC and healthy subjects.

In 2015, Xiao et al. [65] explored the use of methylated NDRG4 gene as a candidate biomarker in urine and stool for diagnosis of CRC. They collected DNA samples from 84 patients and, using nested methylation-specific PCR and denaturing high-performance liquid chromatography (DHPLC), found the mNDRG4 gene to be present in colorectal carcinoma tissue, paracarcinoma tissues, stool, blood, and urine. The sensitivity and specificity of methylated NDRG4 gene expression was analyzed and compared with 16 age-matched healthy controls. In stool, the positive detection rate was 76.2% and in urine 72.6%. Considering the convenience of the acquisition of urine samples, the team collected samples from an additional group of 76 patients with CRC. The positive detection rate of methylated NDRG4 was 72.4% (55/76) in this cohort.

Moreover, a recent study by Song et al. [64] evaluated the sensitivity of total ctDNA recovered from urine and its clinical relevance in diagnosing metastatic CRC. The total DNA quantities in urine specimens of 150 CRC patients were prospectively examined in serial samplings during treatment, and the team found tumor and urine specimens’ molecular profiles to have 90% concordance. Having established a cutoff of 8.15 ng for elevated total DNA, mCRC patients were compared to healthy volunteers and were identified with sensitivity 90.7% and specificity 82.0%. Even if this study did not focus on early CRC diagnosis, it points out how urine total ctDNA could represent a promising diagnostic tool to evaluate in future studies.

## 3. Discussion

This review identified a large number of recent studies exploring non-invasive techniques for diagnosis of CRC. The feature of non-invasiveness acquires fundamental importance when considering recent statistics regarding screening uptake. In the US, around 30–50% of individuals eligible for CRC screening never begin the process and more than half of individuals presenting abnormal results in their initial screening do not complete follow-up investigations [73].

European data in the field are much more fragmented due to the high difference in screening programs between countries. However, statistics show that the overall participation is 49.5% (ranges between 22.8–71.3%) in countries adopting FIT and 33.2% (ranges between 4.5–66.6%) in countries adopting gFOBT, whereas the desirable uptake rate in the EU Guidelines is >65% [2,114].

In sight of this, the ability of these new approaches to achieve an early diagnosis while minimizing stress and discomfort for the patient is noteworthy. In particular, urinary VOCs, exhaled breath VOCs, and saliva miRNAs seem to be the most promising techniques in terms of ease of collection. An important aspect to consider in VOCs-based tests (including stool VOCs-based tests) is that some exploit electronic devices, which use pattern recognition techniques. This technology is portable and inexpensive, making screening simpler and more applicable on a wider scale.

However, even though a number of these studies have reported promising diagnostic performance—comparable to FIT—validation in studies conducted in true screening settings (in an average risk population not undergoing opportunistic screening) would be essential.

As cost-effectiveness is a fundamental characteristic of a good screening program, further research should be dedicated to the cost-effective nature of the previously compiled list of alternative screening techniques with respects to the gold-standards FIT and colonoscopy.

## 4. Conclusions and Future Perspectives

To our knowledge, this review represents the most recent and complete summary of the novel non-invasive screening techniques available in the field of CRC detection. Our observations indicate that to validate the feasibility of most of the alternative tests presented, further studies will be necessary.

In fact, techniques that have already achieved approval from national agencies for drug regulation such as the American FDA, subsequent to studies conducted in true screening settings, appear to be the most reliable. Among them, the blood-based *SEPT9* gene methylation assay is the most promising one, achieving 68.2% sensitivity and 80% specificity using the Epi proColon^®^ 2.0 kit and up to 82% sensitivity and 95.90% specificity using different kits.

In general, gene methylation assays proved themselves to be an interesting diagnostic tool in the field of CRC screening: different methylated gene panels exhibited up to 97% sensitivity and specificity.

Among the other blood based tests described, non-protein-coding RNAs, particularly miRNAs, presented good discriminative ability (up to 89% sensitivity and 93% specificity for detecting CRC and up to 95% sensitivity and 90% specificity for detecting AAs) and therefore could be considered to be included in screening programs.

As a general rule, given that higher adherence rates might be achieved in blood-based compared to stool-based tests, they promise to become good alternatives for CRC screening in the US and Europe as well.

Similarly, the Multitarget stool DNA (MT-sDNA) test Cologuard^®^, already recommended by the American Cancer Society guidelines, represents another feasible option that could catch on in Europe seen as it presents higher sensitivity than FIT (92.3%). As already mentioned in the discussion section, stool, urinary, and exhaled breath VOCs that utilize portable and inexpensive electronic devices based on pattern recognition could make screening simpler and more applicable on a wider scale. However, validation in studies conducted in true screening settings will be essential to determine whether these techniques already present efficacy and features needed to supersede the already in-place screening tests.

In conclusion, because of the ease in sample acquisition, good sensitivities and specificities, and methodology broadly available, Epi proColon^®^ 2.0, Cologuard^®^, and some of the VOCs-based techniques exploiting portable and low-cost electronic devices (e.g., Aeonose^®^, SCENT A1^®^), are likely to enter (or become more common) in clinical practice.

On the other hand, techniques that achieved good diagnostic performances (e.g., miRNA panels) but do not have relevant diagnostic products widely available for clinical use, are less likely to enter clinical practice in the near future.

Moreover, since there are many alternatives that may be worth looking into in the context of true screening programs, a possible approach to avoid exposing a hypothetical target population to higher false negative rates could be to combine the test object of the study to FIT. This method showed positive results, for example, for the m*SEPT9* + FIT combination, which enhanced the sensitivity compared to FIT alone.

## Figures and Tables

**Table 2:** Efficacy of SEPT9 in combination with FIT or CEA for CRC detection.

SEPT 9 Combined with Other Commonly Used Tests	Model(s)	Sensitivity	Specificity	Significant Outcomes and Possible Limitations	References
**SEPT9 + FIT**	Chi-square test or Fisher’s exact test were used to analyze differences in sensitivity. ROC curve evaluated the diagnostic accurance. Comparison among the methods of FIT, mSEPT9, and the combination were evaluated by AUC. Two-side *p* value < 0.05 was considered statistically significant.	84.1%	62.2%	The combination of mSEPT9 with FOBT further improved the AUC value, reaching 0.807 (95% CI 0.752–0.863). The overall sensitivity was 86% for colon and 80.7% for rectum, 100.0% for stage I, 82.6% for stage II, 88.9% for stage III, and 50.0% for stage IV. It aslo reached 85.7%, 83.3%, and 82.4% for patients with regional lymph node metastasis, with distant metastasis, and with vascular and neural infiltration, respectively. However, the combination of the two tests caused a decline in specificity [62.2% (50.8–72.4%)].	[19]
**SEPT9 + FIT**	Data from sensitivity and specificity were used to plot the ROC curve. Because most cycle threshold (Ct) values from normal controls were not detected in the PCR reaction, the Ct value was set to 45 (the maximal number of PCR cycles that ran in the assay) for those non-detected normal controls to plot the curve. This limitation led to the lack of specificity data points for Ct values > 45. Therefore, no data were plotted above certain percentage for 1-specificity (the x axis) in the ROC curves.	94.4%	NA	The combination of SEPT9 with FIT exhibited high sensitivity (94.4%), and the combination of SEPT9, FIT, and CEA increased the sensitivity from 76.6% (SEPT9 alone) to 97.2%. Instead, the combination of FIT + CEA showed no significant difference with SEPT9 alone. The authors concluded that because the contribution of CEA was limited, SEPT9 + FIT alone might be the optimal strategy in CRC opportunistic screening.	[16]
**SEPT9 + CEA**		86.4%	NA		
**SEPT9 + CEA + FIT**		97.2%	NA		

## Supplementary Materials

The following are available online at https://www.mdpi.com/article/10.3390/cancers13081820/s1, Table S1: Efficacy of SEPT9 methylation assays based tests for CRC detection, Table S2: Summary of methylated genes panels feasible for CRC detection, Table S3: miRNAs as CRC biomarkers, Table S4: piRNAs as CRC biomarkers, Table S5: t-M2-PK as CRC screening tool, Table S6: Gut microbiota as CRC screening tool, Table S7: Stool volatile organic compounds (VOCs) based CRC test: chemical analytical and pattern recognition techniques. References [16,17,19,20,21,22,23,27,28,29,30,31,32,33,34,35,43,44,45,46,47,48,49,50,52,53,54,55,56,57] are cited in the Supplementary Materials.
